# The Importance of Caveolin-1 as Key-Regulator of Three-Dimensional Growth in Thyroid Cancer Cells Cultured under Real and Simulated Microgravity Conditions

**DOI:** 10.3390/ijms161226108

**Published:** 2015-11-30

**Authors:** Stefan Riwaldt, Johann Bauer, Jessica Pietsch, Markus Braun, Jürgen Segerer, Achim Schwarzwälder, Thomas J. Corydon, Manfred Infanger, Daniela Grimm

**Affiliations:** 1 Plastic, Aesthetic and Hand Surgery, Otto-von-Guericke University Clinic, Leipziger Str. 44, 39120 Magdeburg, Germany; stefan.riwaldt@med.ovgu.de (S.R.); jessica.pietsch@med.ovgu.de (J.P.); manfred.infanger@med.ovgu.de (M.I.); dgg@biomed.au.dk (D.G.); 2 Max Planck Institute for Biochemistry, Am Klopferspitz 18, 82152 Martinsried, Germany; 3 Institute for Molecular Physiology and Biotechnology of Plants (IMBIO), Gravitational Biology Group, University of Bonn, Karlrobert-Kreiten-Str. 13, 53115 Bonn, Germany; m.braun@dlr.de; 4 Airbus Defense and Space GmbH (ADS), Claude-Dornier-Straße, 88090 Immenstaad, Germany; juergen.segerer@airbus.com (J.S.); achim.schwarzwaelder@airbus.com (A.S.); 5 Institute of Biomedicine, Aarhus University, Wilhelm Meyers Allé 4, 8000 Aarhus C, Denmark; corydon@biomed.au.dk

**Keywords:** international space station, pathway studio, plasminogen, tissue factor, VCAM-1

## Abstract

We recently demonstrated that the *CAV1* gene was down-regulated, when poorly differentiated thyroid FTC-133 cancer cells formed spheroids under simulated microgravity conditions. Here, we present evidence that the caveolin-1 protein is involved in the inhibition of spheroid formation, when confluent monolayers are exposed to microgravity. The evidence is based on proteins detected in cells and their supernatants of the recent spaceflight experiment: “NanoRacks-CellBox-Thyroid Cancer”. The culture supernatant had been collected in a special container adjacent to the flight hardware incubation chamber and stored at low temperature until it was analyzed by Multi-Analyte Profiling (MAP) technology, while the cells remaining in the incubation chamber were fixed by RNA*later* and examined by mass spectrometry. The soluble proteins identified by MAP were investigated in regard to their mutual interactions and their influence on proteins, which were associated with the cells secreting the soluble proteins and had been identified in a preceding study. A Pathway Studio v.11 analysis of the soluble and cell-associated proteins together with protein kinase C alpha (PRKCA) suggests that caveolin-1 is involved, when plasminogen enriched in the extracellular space is not activated and the vascular cellular adhesion molecule (VCAM-1) mediated cell–cell adhesion is simultaneously strengthened and activated PRKCA is recruited in caveolae, while the thyroid cancer cells do not form spheroids.

## 1. Introduction

Poorly differentiated thyroid cancer (PDTC) types have a high risk of local recurrence and exert a missing or insufficient uptake of radioiodine [1,2,3]. A progress of cancer in the course of therapy with polytope metastatic invasion is the indication for external beam irradiation and requires a systemic therapy, because the 10-year survival rate is lower than 15% [4]. Cytostatic chemotherapeutic drugs have a significant toxicity and show only transient and limited response rates [5]. Therefore, the development of new treatment strategies and the search of new target proteins are important topics.

In our approach to this aim, we investigated *in vitro* the migration and aggregation behavior of human thyroid cells including primary tumor cells and cell lines [6,7,8]. Thereby, we learned that culturing the cells above an agarose gel in a 96-well plate (liquid-overlay technique) challenged 3D spheroid formation but prevented monolayer formation [6,7,9]. Moreover, we exposed subconfluent monolayers to real (spaceflight) or simulated microgravity (Random Positioning Machine (RPM)), 2D clinostat) [8,9,10,11]. Under both conditions, the cells separate into two populations of which one remains adherent to the TECAPEEK or plastic surface of the culture dishes, while the other one forms spherical aggregates (multicellular spheroids, MCS) or tubes, which were floating in the culture supernatant [8,9,10,11,12,13].

Challenged by these observations, we became interested in genes and proteins, which might regulate the cellular transition from a two- to a three-dimensional type of growth. As the genetic background of primary cultures scattered very much due to different tumor donors required for the experiments, we used the thyroid cancer cell lines FTC-133 and ML-1 [14,15] to perform molecular studies on thyroid cells which had been exposed to real or simulated microgravity. A number of molecules were detected, which appear to be up- or down-regulated on the proteomic and/or genomic level, when cells were exposed to microgravity [8,9,10,11,12,16,17]. Differences in gene expression patterns and accumulation of proteins were observed, when control cells, which were incubated under normal laboratory conditions (1*g*-samples), were compared with cells forming spheroids under simulated or real microgravity. Our results nicely completed the studies of a number of other researchers evaluated and cited in [13,18].

In addition, we recognized that exposing cells to microgravity did not only change the expression of single dispersed genes or proteins, because we repeatedly observed that groups of interacting genes or proteins were together up- or down-regulated under defined conditions [16,17,18,19,20]. This suggested that several members of whole signaling pathways are changed also in thyroid cells as it was described for lymphocytes, where the protein kinase A (PKA) had been shown to be involved in the loss of T-cell activation in altered gravity using the RPM [21,22] or for fibroblasts, whose microgravity-dependent regulation of various types of collagen and integrins affected MAPK intracellular signaling pathways [23].

Recently, we detected a group of proteins related to the extracellular matrix, when we determined the proteome of FTC-133 thyroid cells, which had been flown to the ISS (Cellbox-1 experiment) and were cultured there for 10 days [24]. In contrast to FTC-133 cells exposed to microgravity in earlier experiments, the cells obtained after the Cellbox-1 mission had not formed spheroids during their stay in microgravity. The obvious reason for this failure was that their exposure to microgravity began after the cell monolayer had reached confluence [10,24]. Hence, the study suggested that FTC-133 cells form spheroids, when sub-confluent monolayers are exposed to microgravity [10,11], but the cells remain adherent, when confluent cell monolayers are exposed to microgravity conditions [24]. The group of proteins mentioned above included caveolin-1. The *CAV1* mRNA was down-regulated during spheroid formation on devices simulating microgravity [11].

In this study, we determined the proteins of the supernatant of the FTC-133 cells, which did not form spheroids during the Cellbox-1 mission [24]. The detected soluble proteins were analyzed by Pathway Studio v11 [25] in regard to their mutual interaction and their effect on the above mentioned group of proteins associated with the cells, which secreted the supernatant proteins, while they did not form spheroids in microgravity. Plasminogen and caveolin-1 attracted special attention, as both have already been described to be involved in anchorage independent growth of cancer cells [26,27].

## 2. Results and Discussion

### 2.1. Proteins in Cell Supernatants

**Figure 1:**
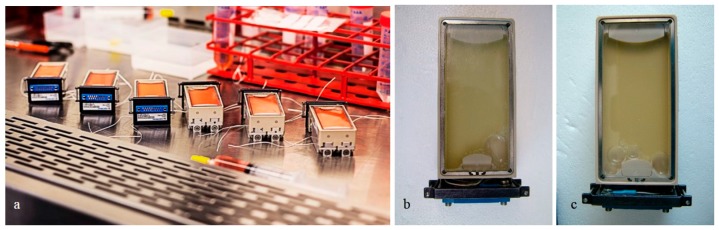
(**a**) Cell suspensions prepared in six spaceflight experiment containers. They were flown to the ISS, and another set was kept on ground as 1*g*-controls; (**b**,**c**) The cell culture chambers of the experiment units containing cell monolayers fixed with RNA*later*.

Cell-associated [24] and secreted proteins of FTC-133 thyroid cancer cells were investigated, after the cells had been cultured in spaceflight experiment containers (Figure 1) either during the Cellbox-1 spaceflight to the ISS including a 10-day-stay on the ISS (r-μ*g*-samples) or in an ISS-like-environment in a ground laboratory room (1*g*-sample). The cells, which did not form spheroids in microgravity, were fixed with RNA*later* and conserved until a proteome analysis was possible, which revealed 29 proteins that have not been detected before in thyroid protein analyses [24].

The supernatants were analyzed by the company Myriad RBM, Austin, TX, USA. The Human Inflammation MAP and the Human Kidney MAP were chosen for the investigation of cytokines. Table 1 gives an overview of the 54 proteins, analyzed by the two MAPs indicated above. Thirty-eight of the searched antigens were not detected. However, antibodies against 16 different proteins made their targets visible, although the values of two remained below the least detectable dose (LDD). They are indicated in Table 1.

IL-6 and IL-8, which are known to be involved in angiogenesis and metastasis in different types of cancer, influence the formation and growth of MCS established under 1*g*-conditions using the liquid-overlay technique [9,28]. They were clearly elevated in space compared with 1*g*-samples (IL-6: 4.46-fold; IL-8 3.65-fold). In addition, the release of IL-7 was significantly elevated in Space samples. Its role in relation to thyroid cancer is still not known. Elevated serum levels of IL-7 in conjunction with IL-6, IL-10 and IL-14 seem to indicate both benign and malignant thyroid disease [29].

In addition, vascular endothelial growth factor (VEGF) was significantly elevated 2.63-fold in Space. VEGF promotes neo-angiogenesis and tumor growth [30,31] and is therefore currently a hot topic in cancer therapy [32,33].

Interestingly, the level of the VEGF secretion was 10-fold higher during the Shenzhou-8 space mission when FTC-133 cells formed large spheroids [10,17], as compared to the Cellbox-1 mission indicating that the highest amount of VEGF was released from FTC-133 cells, which grew in form of 3D aggregates. Moreover, eotaxin-1, osteopontin, NGAL and IL-17 were not released by the FTC-133 cells during the Cellbox-1 spaceflight, although they could be measured after the earlier Shenzhou-8 mission [17] as well as after a 72 h lasting growth under simulated microgravity [11], when IL-7 was not found but spheroids were formed.

**Table 1:** Proteins searched in supernatants of FTC-133 cells with the help of multi-analyte profiling (MAP) technology.

Factor	LDD (pg/mL)	μ*g* ISS	1*g*	Factor	LDD (pg/mL)	μ*g* ISS	1*g*
		(pg/mL)	(pg/mL)			(pg/mL)	(pg/mL)
AAT	58	n.d.	n.d.	IL-10	0.66	n.d.	n.d.
A1M	35	n.d.	n.d.	IL-12p40	18	n.d.	n.d.
A2M	170 ^+^	267 ± 12.5 ^†^	267 ± 12.5 ^†^	IL-12p70	6.7	n.d.	n.d.
B2M	58	1433 ± 309 *	887 ± 266	IL-15	58	n.d.	n.d.
BDNF	5.2	n.d.	n.d.	IL-17	0.42	n.d.	n.d.
CRP	4.2	n.d.	n.d.	IL-18	4.7	4 ± 1 *	2 ± 0
Calbindin	940	n.d.	n.d.	IL-23	80	n.d.	n.d.
CLU	2800	n.d.	n.d.	KIM-1	3.2	n.d.	n.d.
Cystatin-C	20	357 ± 68 *	146 ± 34	MIP-1alpha	2.4	n.d.	n.d.
Eotaxin-1	11	n.d.	n.d.	MIP-1 beta	3.5	16 ± 3 *	9 ± 2
Factor VII	480	n.d.	n.d.	MMP-3	6.6	107 ± 39 *	18 ± 4
FRTN	7.5	2633 ± 1597 *	970 ± 93	MMP-9	3500	n.d.	n.d.
Fibrinogen	43	n.d.	n.d.	MCP-1	15	14 ± 1 *	10 ± 1
GM-CSF	3.5	8 ± 2 *	4 ± 0	NGAL	50	n.d.	n.d.
Haptoglobin	74	670 ± 432 *	13000 ± 1633	Osteopontin	400	n.d.	n.d.
ICAM-1	560	n.d.	n.d.	SCF	17	n.d.	n.d.
IFN-gamma	0.3	n.d.	n.d.	RANTES	0.28	n.d.	n.d.
IL-1 alpha	0.78	n.d.	n.d.	THP	130	n.d.	n.d.
IL-1 beta	0.46	n.d.	n.d.	TIMP-1	6.1	607 ± 69 *	217 ± 52
IL-1 ra	5	n.d.	n.d.	TNF-alpha	5.2	n.d.	n.d.
IL-2	5.7	n.d.	n.d.	TNF-beta	6.4	n.d.	n.d.
IL-3	1	n.d.	n.d.	TNFR-2	1.6	n.d.	n.d.
IL-4	8.4	n.d.	n.d.	TFF3	28	n.d.	n.d.
IL-5	8.7	n.d.	n.d.	VCAM-1	8	127 ± 17 *	52 ± 8
IL-6	1	851 ± 241 *	191 ± 57	VEGF	4.7	561 ± 43 *	213 ± 26
IL-7	6.1	22 ± 2 *	12 ± 4	VDBP	15	n.d.	n.d.
IL-8	0.56	6153 ± 2136 *	1727 ± 482	vWF	780	n.d.	n.d.

Values are given with mean ± SD; 1*g*, corresponding ground control; n.d., not detectable; * *p* < 0.05 for space sample *vs.* corresponding 1*g*-ground control; LDD (Least Detectable Dose)-determined as the mean ± 3 standard deviations; ^†^ unit changed to ng/mL.

We also detected the tissue inhibitor of matrix metalloproteinase 1 (TIMP-1) 2.8-fold and MMP-3 5.94-fold enhanced in space samples compared to 1*g*-samples. This is in contrast to earlier results, when the MMP-3 secretion was found decreased on the RPM and after the Shenzhou-8 spaceflight [17]. It is known that the extracellular matrix (ECM) is very important for tissue maintenance and integrity [34]. MMPs enzymes are involved in ECM degradation and play a role in cancer progression. Their activity is regulated by TIMPs [35]. The role of TIMPs in tumor growth and metastasis is not entirely clear. There are reports of growth-suppressing as well as growth-supporting properties [36]. The release of the vascular cellular adhesion molecule (VCAM-1) was elevated 2.44-fold in Space, whereas the soluble intercellular adhesion molecule (ICAM-1) was not detected in the supernatant of the FTC-133 cells, like during an earlier study [11]. Both play an essential role in the process of thyroid cancer growth [37]. Like IL-18, monocyte chemotactic protein 1 (MCP-1) was detected but remained below the LDD in r-μ*g*- and 1*g*-samples. After the Shenzhou-8 mission (10 days), FTC-133 cells released 8 pg/mL MCP-1 in the spaceflight 1*g*-sample and 6.15 pg/mL MCP-1 in r-μ*g*-samples [17], while under simulated microgravity an up-regulation of MCP-1 was observed in both RPM- and clinostat-samples of ML-1 cells compared with static 1*g-*control samples [9]. Tanaka *et al.* [38] had demonstrated that immunohistochemically staining of papillary thyroid tumor samples indicated a correlation of MCP-1 expression with aggressive behavior of this tumor.

The macrophage inflammatory protein-1 beta (MIP-1β) was increased 1.77-fold in the Space sample, while MIP-1α was not detectable (Table 1). Both forms show diverging signaling [39]. Hence, it is of interest that we did not detect MIP-1α during the Shenzhou-8 spaceflight either, but when FTC-133 cells were cultured under conditions of simulated microgravity [11]. Ferritin was elevated 2.7-fold in r-μg-samples of the FTC-133 cells compared to control samples. The importance of soluble ferritin is still unclear. An abnormal ferritin expression was detected in thyroid and other tumors [40,41]. The release of haptoglobin in space was significantly decreased compared to samples cultured on ground. Fan *et al.* showed that the level of haptoglobin alpha-1 chain (9190 Da) progressively increased with the clinical stages I, II, III and IV in papillary thyroid cancer [42]. The cysteine proteinase inhibitor cystatin-C was 2.44-fold higher released in orbit than on Earth. Its release in the breast cancer tumor microenvironment reduces the proteolytic degradation of extracellular substrates at low pH [43].

In addition, alpha-2-macroglobulin (A2M) was clearly secreted by the thyroid cancer cells on Earth and in space, but the secretion level was not changed. Beta-2-microglobulin (B2M) was highly secreted by the FTC-133 cells (887 pg/mL; LDD was 58 pg/mL) and increased 1.62-fold in space. B2M is involved in biological processes like growth, survival, apoptosis, or metastasis of cancer cells and is currently considered to be a target of tumor treatment with antibodies [44]. GM-CSF is twofold elevated in r-μ*g*-samples. A similar result was found after three days on the RPM [9]. Interestingly, during the Shenzhou-8 mission in 2011 the secretion of GM-CSF was reduced [17] and also when FTC-133 cells were cultured on the 2D-clinostat [11]. GM-CSF is used as an adjuvant in tumor vaccination. Its usefulness, however, is controversial [45].

Taken together the results of Table 1, most of the detected cytokines were differently secreted during the Cellbox-1 space mission as compared to the earlier Shenzhou-8 mission. After the Cellbox-1 mission no spheroids were found, whereas during the Shenzhou-8 spaceflight and the accompanying RPM study [10,24] or in a microgravity simulation device comparison study [11] multicellular spheroids were formed in all experiments. This suggests that a relationship may exist between the proteins detected in the supernatant and the behavior of the cells secreting these proteins during the Cellbox-1 mission, as different types of proteins or equal proteins at different quantities were released into the supernatants, when the FTC-133 cells formed spheroids during the Shenzhou-8 mission [17] and device comparison study [11]. In addition, virtually all the proteins detected in the Cellbox-1 supernatants are of interest in cancer research and medicine.

### 2.2. Interaction of Proteins in Cell Supernatants

**Figure 2:**
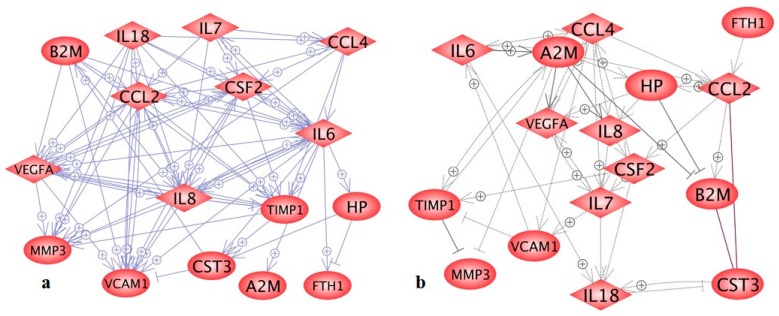
Relationships of the 16 proteins found in the supernatants of the Cellbox-1 experiments (see Table 1). (**a**) Mutual genetic regulation (**b**) Interaction and regulation on the protein level (solid lines indicate binding; arrows indicate direct regulation).

Hence, we studied whether the soluble factors found in the supernatant could influence cellular sheering out of the monolayer under microgravity. For this purpose, we first investigated their mutual interaction by applying the Pathway Studio program. Figure 2 shows the interaction of the secreted proteins at gene (a) and protein levels (b). It can be clearly recognized that all proteins detected belong to a network of mutual interaction. This is true when genes are emphasized (a) and also when proteins are investigated (b). In both cases, mutual up-regulation (+) is dominating.

### 2.3. Interaction of Supernatant and Cell Associated Proteins

**Figure 3:**
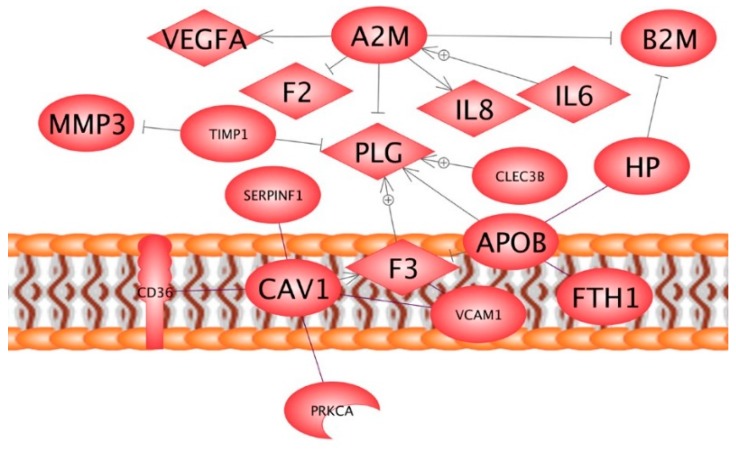
Network of interacting proteins: Lines show a kind of binding like it occurs e.g., when TIMP1 binds to the catalytic domain of MMP3 blocking its proteolytic site, arrows indicate regulation by direct interaction, *i.e.*, by binding which effects conformational or local changes of the target protein, (+) mean up-regulation. The protein names are arranged according to the cellular locations of proteins. Upper part: extracellular proteins, middle part: membrane proteins, lower part (PRKCA): intracellular proteins.

Secondly, we wanted to know whether members of the protein network shown in Figure 2B could have influence on the proteins detected in thyroid cancer cells by mass spectrometry [24], when the FTC-133 cells did not form spheroids under microgravity. For this purpose, we entered these SwissProt numbers together in the Pathway Studio program: (i) SwissProt numbers of the proteins detected by the MAP technology as indicated in Table 1; (ii) SwissProt numbers of the proteins detected earlier in thyroid cancer cells by mass spectrometry [24] and (iii) SwissProt number of PRKCA coded by a gene which showed a slight but insignificant tendency of up-regulation in MCS [12,17]. This program revealed a network of proteins as shown in Figure 3, which comprises 10 of the soluble proteins found in the supernatant and eight of the cell associated proteins determined in the preceding proteome study. In addition, a possible link of the PRKCA protein into the network via caveolin-1 as well as three centers of interaction around A2M, plasminogen (PLG) and tissue factor (F3) became obvious.

The most central point of intersection of this network is plasminogen (PLG). This zymogen is the inactive form of the proteolytic serine enzyme plasmin, which facilitates cancer cell migration as well as spheroid formation [26]. Its activation is tightly regulated by a number of activators and inhibitors [46]. If plasmin is generated, it can activate more plasminogen creating a plasmin amplification spiral [26]. Alpha-2-macroglobulin interrupts this loop by inactivating plasmin directly [26,47]. In the MAP analysis described, we found A2M for the first time, but still at low concentration (50% above LDD). Equal amounts of this soluble protein were secreted by cells in r-μ*g*- and 1*g*-samples, although more B2M, IL-6, IL-8 and VEGFA were found in supernatants of r-μ*g*-samples (Table 1). Hence, its inhibitory influence on plasminogen activation may be equal under both conditions. In addition, TIMP-1 reduces activation of plasminogen by inhibiting plasminogen activators and metalloproteinases [48]. It was enhanced in flown samples, but MMP-3 was enhanced even more. Therefore, the inhibition of PLG activation by TIMP1 in r-μ*g*- and 1*g*-samples cannot be estimated now. Furthermore, apolipoprotein B (APO-B) is inhibiting the fibrinolytic activity of plasminogen, *i.e.*, its activation [49]. Its concentration could not be quantified in the MS analysis. However, the fact that it was only detectable, when FTC-133 cells did not form spheroids strongly points to its inhibitory effects.

Tetranectin (CLEC3B) is also a member of the network. It binds to plasminogen and appears to function as an anchor or reservoir of this protein [50]. Tetranectin bound to plasminogen inhibits also its binding to the extracellular matrix, where plasmin causes degradation [51,52]. F3 (tissue factor) exerts stimulatory effects on plasminogen activation [53]. This effect is inhibited by complex formation with APO-B [54]. Tissue factor resides in various compartments of a cell. At the cell surface, it is co-localized with caveolin-1 [55]. Dependent on the kind of insertion in caveolae, caveolin-1 may expose the tissue factor to its pathway inhibitors [56]. Hence, it seems that down-regulation of plasminogen activation during the Cellbox-1 spaceflight could have prevented spheroid formation [26].

Caveolin-1 also supports cell-cell adhesion via membrane-bound VCAM-1. If caveolin-1 expression is knocked down by siRNA, VCAM-dependent cell-cell adhesion is blocked [57]. We only found the soluble version of VCAM-1 in the Cellbox-1 study. However, the finding indicates that VCAM-1 was highly expressed and even enhanced in the r-μ*g*-samples. Caveolin-1 also forms scaffolds, which regulate the activity of PRKCA by its appropriate insertion in caveolae [58]. Taken together, the proteins shown in Figure 3 appear to contribute in preventing spheroid formation under microgravity, when the cells have reached a complete confluence at exposure-start. Caveolin-1 appears to play a central role in this game.

### 2.4. Possible Mechanisms of the Inhibition of Spheroid Formation

Therefore, we looked closer at caveolin-1 and searched through further proteins that may interact with it. Of these proteins, we selected those which were earlier detected in our microgravity research projects.

**Figure 4:**
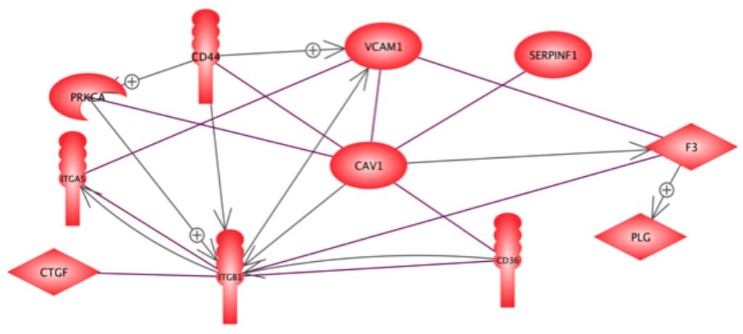
Proteins binding to and interacting with caveolin-1.

Figure 4 indicates seven proteins, which might regulate spheroid formation and simultaneously can bind to caveolin-1. In this manuscript, we explain how caveolin-1 interacts with the tissue factor (F3) and may contribute together with TIMP1, A2M and APO-B to the inhibition of plasminogen activation so that plasmin is not accumulated to such a concentration that it could trigger spheroid formation, even when this process is supported by real microgravity [10,26]. In addition, caveolin-1 strengthens the cell-cell adhesion mediated by VCAM-1 [57]. Thereby, it may prevent the cells sheering out of the monolayer. VCAM-1 also interacts with integrin-alpha-5 (Figure 4). Interestingly, FTC-133 cells, in which integrin-alpha-5 was found in a preceding proteome study, form faster large spheroids than CGTH-W-1 thyroid cells, in which integrin-alpha-5 could not be detected [59].

In a preceding gene analysis with FTC-133 cells exposed to simulated microgravity, one part of the cells formed spheroids, while the other part continued to grow as monolayer. The *CD44* and *ITGB1* (integrin-beta-1) genes were only up-regulated within the population of adherent cells [12]. Furthermore, *CAV-1* and *CTGF* genes exerting proteins related via integrin-beta-1 (Figure 4) were simultaneously down-regulated in those cells forming MCS [11]. It remains to be determined whether these two genes are also down-regulated when confluent monolayers are exposed to simulated microgravity.

Although the impact of caveolin-1 binding to CD36 and SERPINF1 still has to be investigated, it seems that caveolin-1 inhibits anchorage independent cell growth of cancer cells [27] initiating several cascades of protein reactions. Further spaceflight experiments on up- and down-regulation of caveolin-1 appear to be worthwhile, because several studies in cancer research revealed a role of this protein in tumor cell development. In normal breast epithelial cell lines (12N, MCF10), but not in breast cancer cell lines like MCF7 or T47D, *CAV* mRNA and caveolin proteins were found [60]. A similar observation was made for caveolin-1, when bronchial epithelial cell lines were compared with lung cancer cell lines [61]. Furthermore, immunohistochemical analyses of human ovarian tissue specimens revealed expression of the protein in normal tissue but not in serous ovarian carcinomas [62]. Interestingly, cancer cells lost their capability of anchorage independent growth, when expression of caveolin-1 was re-induced by suitable vectors [60,63]. Thus, the FTC-133 cells lost their capability to form spheroids, when caveolin-1 protein became detectable in these cells by mass spectrometry, after they had formed confluent monolayers [24]. Hence, space experiments like the one described in this and a preceding paper [24] could be a model to study a cancer suppressive role of caveolin-1.

However, when caveolin-1 expression was studied immunohistochemically on paraffin-embedded bladder tumour sections of 89 patients, only specimens of nine patients with high-grade bladder cancer were caveolin-1-positive [64]. This discrepancy might be explained considering that caveolin-1 not only is capable to strengthen the cell–cell- or cell–ECM binding e.g., via VCAM-1 or integrin-alpha-5 [57,59], but also scaffolds proteins involved in the Ca^2+^ household. Thereby, it could participate in the Ca^2+^ regulation [65]. A Ca^2+^ dependent protein, which binds to caveolin-1 is the protein kinase C-alpha [66,67] (Figure 3). It influences the cytoplasmic Ca^2+^ oscillation [68], whose frequency has effects on gene expression [69]. As far as we know, an influence of Ca^2+^ regulation on microgravity related, scaffold-free spheroid formation has not yet been described. Future studies may show, whether Ca^2+^ regulation mediates the changes of the expression of the genes, whose products are involved in microgravity-dependent spheroid formation of thyroid cells [70]. They could make spaceflight experiments on cancer cells even more meaningful.

## 3. Experimental Section

### 3.1. Cell Cultures

We cultured poorly differentiated human follicular thyroid cancer cells (FTC-133 cell line) in RPMI-1640 medium supplemented with 10% fetal calf serum (Merck Millipore, Berlin, Germany), penicillin (100 U/mL) and streptomycin (100 μg/mL; Merck Millipore) at 37 °C and 5% CO_2_ as described earlier. We purchased the FTC-133 cells from the Health Protection Agency Culture Collections (HPACC; Salixbury, UK). Prior to use, 10^6^ cells were suspended in freshly prepared culture medium until transferring them into an automated cell culture system constructed for spaceflights [10].

### 3.2. Cellbox-1 Spaceflight Experiment

The Cellbox-1 spaceflight experiment was carried out as described by Riwaldt *et al.* [24]. Shortly after, the launch of the rocket took place on 18 April 2014 from KSC, Cape Canaveral, FL, USA. On 20 May 2014, the cells returned to Earth with the Dragon capsule. The Dragon capsule splashed down into the Pacific near by the Californian coastline. Afterwards, they were delivered to the Space Life Science Laboratories (SLSL), 505 Odyssey Way, Merritt Island, FL, USA [24].

The cells were prepared and treated as described recently [24]. Five days before the rocket launch, we transferred 1 million thyroid cancer cells in each of the experiment containers. We sent six containers to real μ-gravity on board the ISS, whereas three containers, identically in construction, stayed in an ISS-like-environment in a ground laboratory. The cells, which had been sent to space were called r-μ*g*-samples, the ground controls were called 1*g*-samples. Due to a launch delay, a medium exchange became necessary two days before the planned launch date.

### 3.3. Cell Fixation and Medium Exchange

Cell fixation and medium exchange were performed in ground and flight modules in exactly the same manner. The procedure was published in Riwaldt *et al.* [24]. Then, after a total period of 12 days, the pumps integrated in the space-suitable incubation modules worked automatically, replacing the used medium by new medium. Used medium was stored for supernatant analysis. The next step was the cell fixation of FTC-133-cells by RNA*later* with the help of the automatically working pump.

### 3.4. Cytokine Measurements by MAP-Technology

In order to investigate the release of cytokines, we used Multi-Analyte Profiling (MAP) as previously described [11,24]. The MAP-analyses were performed by the company Myriad RBM (Austin, TX, USA). Cell supernatants were investigated with the Human InflammationMAP^®^ and Human KidneyMAP^®^.

### 3.5. Pathway Studio Analysis

Pathway Studio v11 was purchased from Elsevier Research Solutions, Amsterdam, The Netherlands. This program was used online [25]. To start an analysis, the SwissProt numbers of the proteins of interest were entered.

### 3.6. Statistical Evaluation

SPSS^®^ Statistics 23.0 was used for statistical evaluation. Detected cytokine concentration was compared for 1*g* and r-μ*g* using one-way ANOVA. All data are presented as mean ± standard deviation (SD). The significance level was set by *p* < 0.05, * indicating significant differences.

## 4. Conclusions

From our results, we conclude that inhibition of spheroid formation under microgravity and inhibition of anchorage independent growth of various types of cancer cells on the ground proceed via similar pathways [24,26,27]. In both cases, plasminogen and caveolin-1 appear to play a key role. At least concerning the inhibition of spheroid formation under microgravity VCAM, tissue factor, alpha-2-macroglobulin, apolipoprotein B, tissue inhibitor of metalloproteinases-1 and protein kinase C alpha seem to be further members of relevant pathways, like those which regulate plasminogen activation or cell–cell adhesion.

In addition, our approach to focus on proteins, which become detectable only after special treatment, led to valuable information.

## Abbreviations

μ*g*	Microgravity
1*g*-samples	Cells treated and incubated on ground equally as the space-flown cells
r-μ*g*-samples	Cells flown to the ISS
2D	Two-dimensional
3D	Three-dimensional
A2M	Alpha-2-macroglobulin
ANOVA	Analysis of variance
APO-B	Apolipoprotein B
B2M	Beta-2-microglobulin
CAV	Caveolin
CCL	C-C motif chemokine
CD	Cluster of differentiation
CD36	Platelet glycoprotein 4
CLEC3B	Tetranectin
CSF	Colony-stimulating factor
CTGF	Connective Tissue Growth Factor
DLR	Deutsches Zentrum für Luft- und Raumfahrt, German Space Agency
ECM	Extracellular matrix
F2	Prothrombin
F3	Thromboplastin, tissue factor
F3	Tissue factor
FTC	Follicular thyroid cancer
FTH	Ferritin heavy chain
GM-CSF	Granulocyte-macrophage-colony-stimulating factor
HP	Haptoglobin
IL	Interleukin
ISS	International Space Station
ITGB	Integrin beta
KSC	Kennedy Space Center
LDD	Lowest detectable dose
MAP	Multianalyte Profiling
MAP^®^	Multi-Analyte Profiling
MAPK	Mitogen-activated protein kinase
MCP-1	Monocyte chemotactic protein 1
MCS	Multicellular spheroids
MIP-1ß	Macrophage inflammatory Protein 1 beta
MMP-3	Matrix metalloproteinase 3
NASA	National Aeronautic and Space Administration
NGAL	Neutrophil gelatinase-associated lipocalin
PDTC	Poorly differentiated thyroid cancer
PDTC	Poorly differentiated thyroid cancer cells
PKA	Protein kinase A
PRKCA	Protein kinase C alpha
PLG	Plasminogen
RPM	Random Positioning Machine
RPMI	Roswell Park Memorial Institute medium
SD	Standard deviation
SERPIN	Serin protease inhibitor
SLSL	Space Live Science laboratories
SPSS^®^	Statistical Package for the Social Sciences
TIMP-1	Tissue inhibitor of matrix metalloproteinase 1
VCAM-1	Vascular cellular adhesion molecule-1
VEGF	Vascular endothelial growth factor
